# Supplementary material for: Stacking Model‐Based Classifiers for Dealing With Multiple Sets of Noisy Labels
Source: Biom J. 2025 Mar 12;67(2):e70042. doi: 10.1002/bimj.70042 (PMC11898607; doi:10.1002/bimj.70042)
Supplement: Supplementary file 1 — Supporting Information [file BIMJ-67-e70042-s001.zip › density-based-ensemble-model-main/competitors_real_data/netlab-master/netlab-master/help/rbf.htm]

Netlab Reference Manual rbf


# rbf

## Purpose

Creates an RBF network with specified architecture

## Synopsis

```
net = rbf(nin, nhidden, nout, rbfunc)
net = rbf(nin, nhidden, nout, rbfunc, outfunc)
net = rbf(nin, nhidden, nout, rbfunc, outfunc, prior, beta)
```

## Description

`net = rbf(nin, nhidden, nout, rbfunc)` constructs and initialises
a radial basis function network returning a data structure `net`.
The weights are all initialised with a zero mean, unit variance normal
distribution, with the exception of the variances, which are set to one.
This makes use of the Matlab function
`randn` and so the seed for the random weight initialization can be
set using `randn('state', s)` where `s` is the seed value. The
activation functions are defined in terms of the distance between
the data point and the corresponding centre. Note that the functions are
computed to a convenient constant multiple: for example, the Gaussian
is not normalised. (Normalisation is not needed as the function outputs
are linearly combined in the next layer.)

The fields in `net` are

```
  type = 'rbf'
  nin = number of inputs
  nhidden = number of hidden units
  nout = number of outputs
  nwts = total number of weights and biases
  actfn = string defining hidden unit activation function:
    'gaussian' for a radially symmetric Gaussian function.
    'tps' for r^2 log r, the thin plate spline function.
    'r4logr' for r^4 log r.
  outfn = string defining output error function:
    'linear' for linear outputs (default) and SoS error.
    'neuroscale' for Sammon stress measure.
  c = centres
  wi = squared widths (null for rlogr and tps)
  w2 = second layer weight matrix
  b2 = second layer bias vector
```

`net = rbf(nin, nhidden, nout, rbfund, outfunc)` allows the user to
specify the type of error function to be used. The field `outfn`
is set to the value of this string. Linear outputs (for regression problems)
and Neuroscale outputs (for topographic mappings) are supported.

`net = rbf(nin, nhidden, nout, rbfunc, outfunc, prior, beta)`,
in which `prior` is
a scalar, allows the field `net.alpha` in the data structure
`net` to be set, corresponding to a zero-mean isotropic Gaussian
prior with inverse variance with value `prior`. Alternatively,
`prior` can consist of a data structure with fields `alpha`
and `index`, allowing individual Gaussian priors to be set over
groups of weights in the network. Here `alpha` is a column vector
in which each element corresponds to a separate group of weights,
which need not be mutually exclusive. The membership of the groups is
defined by the matrix `indx` in which the columns correspond to
the elements of `alpha`. Each column has one element for each
weight in the matrix, in the order defined by the function
`rbfpak`, and each element is 1 or 0 according to whether the
weight is a member of the corresponding group or not. A utility
function `rbfprior` is provided to help in setting up the
`prior` data structure.

`net = rbf(nin, nhidden, nout, func, prior, beta)` also sets the
additional field `net.beta` in the data structure `net`, where
beta corresponds to the inverse noise variance.

## Example

The following code constructs an RBF network with 1 input and output node
and 5 hidden nodes and then propagates some data `x` through it.

```
net = rbf(1, 5, 1, 'tps');
[y, act] = rbffwd(net, x);
```

## See Also

`rbferr`, `rbffwd`, `rbfgrad`, `rbfpak`, `rbftrain`, `rbfunpak`

---

**Pages:**
Index


---

Copyright (c) Ian T Nabney (1996-9)
